# Science Star over Asia

**DOI:** 10.1371/journal.pbio.0030322

**Published:** 2005-09-13

**Authors:** Chris Y. H Tan

## Abstract

The founding director of Singapore's Institute of Molecular and Cell Biology illustrates the rise of science in Asia.

Recent news of human embryonic stem cell (hESC) research in Seoul has made headlines around the world. In 2004, Korea took the world by surprise when W. S. Hwang and his team published the isolation of hESCs from a cloned blastocyst [1]. One year later, the same Korean team published the establishment of 11 hESC lines, made from transplanting the nucleus of patients' skin cells into donated human oocytes [2]. But few in the Western science community are aware that hESC research has its root in Asia. Ariff Bongso and his associates at Singapore's National University Hospital in 1994 were the first to derive hESCs from a five-day-old discarded human embryo and discover that these cells were pluripotent and, henceforth, had therapeutic potentials as cell transplants [3]. Eight years later, they were able to substitute the use of mouse feeder layer cells with human cell feeders and human serum to grow hESCs, reducing the risk of introducing mouse and bovine pathogens [4] when hESCs are transplanted into patients.

Grit and imagination drove Asian science to compete with the best genomic science in the world.

The stem cell success in Korea comes out of a history in and growing investment in Asia–Pacific science that the West is slowly appreciating. It also comes at a time when independent events in the United States are having an impact on clinical research and health care. The ongoing debate of the Right to Life movement versus allowing hESC research to fight life-threatening diseases continues to polarize public opinion. The recent withdrawals of rofecoxib (Vioxx), methylphenidate (Ritalin), and a few other high-profile drugs from the market threatens the reputation of the pharmaceutical industry. Medicine of the 21st-century is at a crossroad, with immense changes ahead that could change the face of the pharmaceutical and health-care industry. With these changes, Asia is becoming a player in research outsourcing to discover new medicine. Amazingly, for a latecomer to modern drug development, there are now 140 drugs in China's pipeline alone, 60 (10% of the world's total) of which are biologically derived agents, including antibodies and vaccines. And in the near future, it is likely that numerous clinical trials will be performed in Asia as clinical trial costs escalate in the West. This rise of science in the Asia–Pacific region is not simply fortuitous. As I will discuss, it is the result of concentrated efforts to capitalize on its strengths and to form strategic partnerships.

## Traditional Chinese Medicine and Natural Product Drug Discovery

Traditional Chinese medicine (TCM) became a national health-care delivery focus when China became the People's Republic of China in 1949. Medicinal factories were set up to extract TCMs from herbs and to manufacture TCM pills and medicinal powders for China's masses. It took another two decades, during Nixon's Ping-Pong Diplomacy, for the US to discover the wide use of acupuncture in China to treat chronic pain. But until very recently, TCM has remained an enigma for the West. In the 1980s Hong Kong became interested in TCM as a focus for biotechnology. Today Hong Kong researchers collaborate with their Chinese counterparts to search for herbs with medicinal properties by investigating ancient Chinese medical literature, and then finding the described herbs in the collection of herbal libraries in China (Table 1). For instance, in the search for compounds to treat pain, a Chinese biotechnology company (International Wex Technologies) has successfully applied the TCM principle of using one poison to counter another—they have used sublethal doses of tetrodotoxins for the treatment of pain associated with heroin withdrawal [5] and for the management of pain in patients with cancer [6].

**Table 1:**
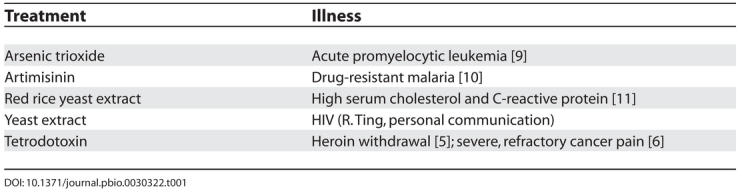
Treatments Derived from TCMs

Most of the active ingredients in TCM are uncharacterized, and its alleged efficacy depends on multiple active ingredients from herbal extracts, whereas Western medicine often relies on the reductive principle of one active ingredient to treat a given disease. But Western medicine of late has also had success in extending the practice of combinatorial drug therapy from cancer treatment to the combination of various anti-HIV drugs to inhibit the rapidly mutating virus in patients with HIV, and the combination of antibiotics and inhibitors of acid secretion for the treatment of gastrointestinal ulcers. It would appear that Western medicine and TCM are beginning to share some common ground in the use of more than one medicine to treat a disease.

In 1993, cell signalling research at Singapore's Institute of Molecular and Cell Biology (IMCB), where I served as the founding director, attracted GlaxoWellcome to set up a High-Throughput Drug Discovery Center in the IMCB to screen for lead compounds from natural products. This collaboration eventually spun off a drug discovery company, MerLion Pharmaceutical, a Singapore-based company that has the world's most comprehensive and diverse collection of natural products (600,000). MerLion is collaborating with several companies such as Abbott Laboratories, Merck, Athelas, NovImmune, Schering–Plough, KuDOS Pharmaceuticals, Fujisawa Pharmaceuticals, and Dow AgroSciences to search for lead compounds in its impressive collection of natural products. High-throughput screening, selection of highly specific cell-signalling intervention points, and computational indexing of libraries cover the value chain of modern drug discovery, and have been established for several years in Singapore. This is now bringing in companies from around the world to the Asia Pacific to discover new leads from natural products.

## Genomics, Gene Therapy, and hESCs

As leadership in Singapore, Hong Kong, Taipei, and Seoul were considering avenues of biotechnology investment in the 1980s, the US and Europe had already decided to sequence the human genome. Apart from Japan, the Asia–Pacific region was generally unprepared for the coming of the age of genomics. By the 1990s, different pieces of the human genome were assigned to different countries for sequencing, with the US having the lion's share. Nevertheless, Singapore's IMCB resolved to make a meaningful contribution to genomics by sequencing the genome of the Japanese puffer fish Fugu rubripes. With such a small genome (365 megabases) and relatively little repetitive DNA, the puffer fish offered a potential tool to annotate and thereby study the human genome. Indeed the puffer fish genome sequence enabled the Singapore-led team to discover approximately 1,000 human putative genes that had not been described in the public annotation prediction databases [7].

In the same year, another genome feat was being accomplished in China, where two US-trained scientists, Gane Wong and Jun Yu, persuaded the Chinese Academy of Sciences to support the genomic sequencing of the most important agricultural plant in the history of Asia, rice. Wong and Yu established the Beijing Genomics Center and gathered 500 young researchers to sequence the rice genome. To beat the competition, it was not uncommon for young researchers to work around the clock, camping in the laboratory. And in 2002, the Beijing Genomics Center surprised the world by announcing that it had sequenced the rice genome [8]. With essentially no track record in the field, IMCB and the Beijing Genomics Center made significant breakthroughs, despite being late off the starting block in the genomics race.

Grit and imagination drove Asian science to compete with the best genomic science in the world. The same can be said of both human stem cell and gene therapy research in Asia, except that the impact is even bigger. The first licensing of a made-in-China gene therapy product surprised the world. SiBiono Gene Technologies, a gene therapy company in Shenzhen, announced the world's first gene therapy medicine, developed using the recombinant adenovirus-p53 tumour suppressor gene for the treatment of head and neck squamous-cell carcinoma. In clinical trials, 64% of patients with late-stage head and neck cancer experienced complete regression of their tumours. SiBiono's success was attributed to developing the right system for delivery of its adenoviral vector. It addressed safety by careful dosing and follow-up of the patients for up to five years.

China's large patient population base means it is easier to recruit patients and, hence, generate clinical data quickly. Clinical trials of gene therapy have not been delayed in China as they were in the West because of a few fatalities in the US and Europe during the initial clinical studies of gene therapy. So far, no deaths have been reported from gene therapy trials in China. The approval of the first gene therapy product has propelled China to the forefront of gene therapy medicine. There are now ten gene therapy products in development in China, compared to 43 in the US and ten in Europe. Patients with head and neck cancer from the US and Europe are opting to go to China for gene therapy treatment. No doubt the rest of the world will closely monitor China's experience with gene therapy when the interests of patients worldwide are at stake.

## Can't Do, Me Too, or Can Do Economies

Some 20 years ago, few scientists in the West would have believed that good science could come out of Asia within their lifetime, since none of the Asian countries except Japan had invested much into basic biological sciences. Only in the early 1980s did the tiger economies of Singapore, Hong Kong, South Korea, and the Republic of China ponder the merit of graduating from a “me too” manufacturing economy to a “can do”, knowledge-based society. Could it be done, and, if so, how long would it take for these countries to build their respective critical mass of scientists?

At that time, Deputy Prime Minister of Singapore, Dr. Goh Keng Swee, the architect of the Singapore economic miracle, asked whether Singapore could build an equivalent of the Weizmann Institute, a state-funded center for scientific excellence. To address this question, the Singapore IMCB was set up in 1987, and it began recruiting young scientists from Europe, the US, and Canada. Within ten years, the IMCB established an infrastructure for intensive research in modern biology (http://www.imcb.a-star.edu.sg). It recruited 300 scientists working on cell signalling mechanisms during differentiation, proliferation, development, cell movement, and host–virus interactions. In as little as one decade, good science was fostered from essentially nothing. Within another decade, the IMCB experiment was expanded to a dozen more research institutes focusing on translation medicine, genomics, neuroscience, nanotechnology, bioinformatics, imaging, drug discovery, infectious diseases, and human disease model systems, all housed in the newly built science city Biopolis, as well as in universities and hospitals. The number of scientists working in Singapore has more than doubled to 3,000 in the past ten years, reflecting an apparent emigrational trend of scientists from the West to the Asia–Pacific region.

If the present is a glimpse of the future, there are several signs of things to come: Alex Matter, the discoverer of the well-known anticancer drug imatinib (Gleevec) has established the Novartis Institute for Tropical Disease Research in Biopolis; David Lane, the discoverer of p53 as a tumour suppressor, has been recruited to succeed myself as Director of IMCB; Sydney Brenner has great enthusiasm to get the Institute of Translation Research going in Singapore; and others like George Radda (United Kingdom), Axel Ullrich (Germany), and K. C. Nicolaou (US) are setting up laboratories in Singapore, while continuing to maintain their main laboratory in the US and Europe. Successful senior Asian scientists in the US such as neuroscientist M. M. Poo at the University of California at Berkeley, X. D. Wong at the University of Texas Southwestern, Yale University's geneticist T. Xu, Singapore IMCB's P. Li, C. W. Wong of Scripps Research Institute, and M. Lai of the University of Southern California have all recently set up corresponding research institutes in China or Taiwan (Taipei).

Movement is also occurring within Asia. In 2001, the recruitment of a leading cancer researcher at the University of Kyoto, Y. Ito, with his entire team to Singapore's IMCB created a debate in the Japanese news media. Shortly after Ito's move to Singapore, Japan's Minister of Science made a serious effort to accelerate reform of modern biology in Japan, an exercise its former Minister of Education started 20 years ago. Although Japan annually invests as much as the US on a per capita basis in biomedical research, by several measures, it is a distant third in the world of modern biology. The decision was made to build a graduate university for modern biology in Okinawa. The president of this graduate university shall be an eminent scientist from outside of Japan; the faculty and the students will consist of a cosmopolitan mix of non-Japanese and Japanese researchers, and the language of instruction will be English. The purpose is to reform modern biology in Japan by enticing promising scientists into Japan through Okinawa. Will this be a successful experiment for Japan even though some consider Okinawa too far away from the centers of excellence on the main island of Japan?

Good science will flourish wherever the conditions are most friendly, as it did in the US in the last century. Many factors, such as good funding, cost per discovery, the ready availability of a large pool of young talent, cultural acceptability of non-natives to Asian societies, and good living conditions must be in place. As knowledge-based societies become a reality, industry worldwide will outsource discovery to wherever the job can be done well and at the lowest cost. In the 20th century, the US was the Mecca of science, attracting the giants of science from the rest of the world. Economic conditions are different today; there is abundant wealth and young talent in Asia. For Asia to become a Mecca of science in the 21st century, countries in the region need to take a long-term view and be as eager to promote scientific collaboration with each other as they are to collaborate with the US.

Without a doubt, the science star is shining over Asia, but Asian science has a glaring deficiency in being highly “Balkanized”. In Europe, support of research crosses national boundaries and is facilitated by the European Molecular Biology Organization. Today research funding crosses few national borders in Asia. Notwithstanding this, eight years ago a small group of visionary scientists led by Kenichi Arai, the former Director of the Institute of Medical Sciences at the University of Tokyo, formed the equivalent of the European Molecular Biology Organization in Asia called the Asia–Pacific International Molecular Biology Network. To date the Asia–Pacific International Molecular Biology Network has 300 scientists in its network, representing key scientists from 16 countries in Asia. In spite of a lack of cross-border funding, the Asia–Pacific International Molecular Biology Network has managed to conduct workshops and symposia annually through innovative fundraising schemes. Good science is on the move in Asia, and when Asia's talents are mobilized, it will move the world as never seen before.

## Abbreviations

IMCB: Institute of Molecular and Cell Biology
hESC: human embryonic stem cell
TCM: traditional Chinese medicine
